# Frontiers in thoracic oncology: new breakthroughs in molecular targets and immunotherapy

**DOI:** 10.3389/fimmu.2025.1721638

**Published:** 2025-12-15

**Authors:** Yujing Yang, Dan Pu, Xuehan Li

**Affiliations:** 1 Department of Anesthesiology, West China Hospital, Sichuan University, Chengdu, China; 2 Lung Cancer Center, West China Hospital, Sichuan University, Chengdu, China

**Keywords:** thoracic tumors, molecular targeted therapy, immunotherapy, treatment progress, immune cell communication

## Abstract

Thoracic tumors, including lung cancer, breast cancer, and thymoma, usually present poor outcomes. The current treatment methods for thoracic tumors have low efficacy and are associated with severe adverse reactions. Molecular targeted therapy and immunotherapy offer new possibilities for the treatment of thoracic tumors. In this review, we have summarized the latest research on these novel therapeutic strategies, and discussed their clinical applications, challenges, and possible countermeasures. This review offers a theoretical basis for improving the outcomes of thoracic tumor patients, along with future research prospects.

## 1 Introduction

Thoracic tumors include lung cancer, breast cancer, esophageal cancer, and thymoma, and are associated with high incidence and mortality rates worldwide. In the present review, the term “thoracic tumors” is used in a deliberately broad and clinically oriented sense to encompass malignancies arising in organs within or immediately adjacent to the thoracic cavity, specifically lung cancer, breast cancer, esophageal cancer and thymoma. Although breast cancer is not invariably classified along with other thoracic malignancies, its anatomical location and the substantial overlap in systemic treatment strategies – particularly regarding molecular targeted therapy and immunotherapy – provide a clear rationale for its inclusion within the scope of this article. Surgical resection, chemotherapy, and radiotherapy are effective in the early stages of thoracic tumors (1, 2), and may achieve clinical cure in a small percentage of patients. However, the curative effects are limited for patients with advanced or metastatic disease (3–5). In addition, the conventional treatments for thoracic tumors are associated with significant adverse reactions (6, 7), which lower the quality of life of patients.

In recent years, novel molecular targeted therapies and immunotherapies have emerged for various thoracic tumors (8–10). Targeted therapies aim at blocking specific receptors or molecules on tumor cells and inhibiting the downstream signaling pathways (11, 12), which selectively eliminate tumor cells while sparing healthy cells. On the other hand, immunotherapies prevent immune evasion and mobilize the host immune surveillance to target and kill tumor cells (13–15). Both therapeutic modalities have significantly improved the curative rate for thoracic tumors and broadened the treatment opportunities for patients (16, 17). In this review, we have discussed the latest progress in molecular targeted therapy and immunotherapy for thoracic tumors, with the aim of providing references for clinical practice and future research (18–20).

Several meta-analyses and umbrella reviews recently published in peer-reviewed oncology journals have systematically quantified the clinical efficacy and safety of molecular targeted agents and immune checkpoint inhibitors (ICIs) against thoracic malignancies. However, these quantitative analyses are typically organ-specific or modality-restricted, and provide limited information regarding the mechanistic basis of treatment response, cross-disease comparisons, and the rational design of combination strategies. In contrast, the present narrative review adopts an integrative perspective across major thoracic tumors, including lung cancer, breast cancer, esophageal cancer, and thymoma. We have not only summarized the latest advances in targeted therapies and immunotherapies, but also the impact of these interventions on tumor cell signaling and the tumor immune microenvironment, as well as the convergent principles that can guide the development of combined targeted-immunotherapy regimens. Furthermore, we have discussed practical challenges such as resistance, toxicity, costs, and biomarker-driven patient selection, and outlined future directions involving multi-omics profiling, liquid biopsy, and artificial-intelligence (AI)-assisted decision-making. By positioning thoracic oncology within this broader mechanistic and translational framework, our review complements and extends existing meta-analyses and umbrella reviews, and aims to provide clinicians and researchers with cross-cutting insights for therapeutic optimization.

## 2 New progress in molecular-targeted therapy

### 2.1 Lung cancer

**Figure 1:**
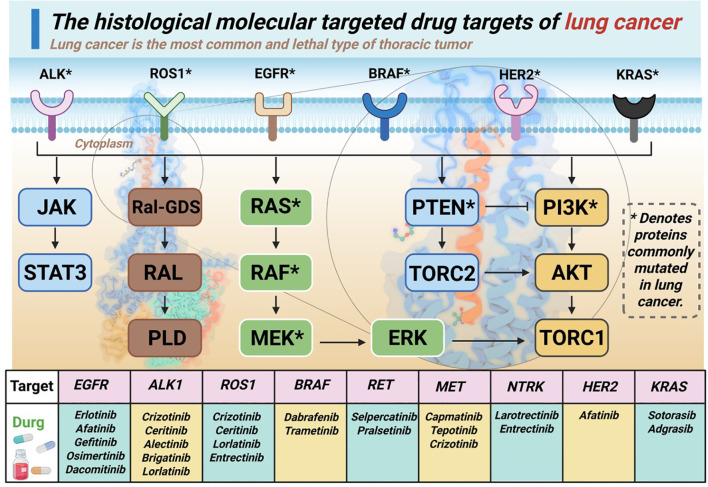
Molecular targets of lung cancer and the corresponding drugs.

Lung cancer is the most prevalent and lethal type of thoracic tumor. According to histological characteristics, lung cancer can be classified as non-small cell lung cancer (NSCLC) and small cell lung cancer (SCLC), with NSCLC accounting for approximately 85% of all cases (21, 22). The treatment of NSCLC has evolved significantly in recent years with the continuous development of drugs targeting different driver gene mutations (**Figure 1**) (23–25).

#### 2.1.1 Drugs targeting epidermal growth factor receptor

EGFR, a member of the receptor tyrosine kinase family, is mutated in various cancers, and the abnormal activation of its downstream signaling pathway plays an important role in tumor cell proliferation, survival, migration, and angiogenesis (26, 27). In fact, EGFR gene mutations are present in almost 30%-50% of the NSCLC patients of Asian ancestry. First-generation EGFR tyrosine kinase inhibitors (TKIs), such as gefitinib and erlotinib, reversibly bind to and inhibit the tyrosine kinase domain of EGFR, thus blocking the downstream RAS-RAF-MEK-ERK and PI3K-AKT-mTOR pathways (28–30), and inhibiting tumor cell proliferation (31, 32). Compared to traditional chemotherapy, first-generation EGFR-TKIs can effectively improve the objective response rate (ORR) and progression-free survival (PFS) of NSCLC patients with EGFR mutations, and are also associated with fewer adverse reactions. However, most patients develop drug resistance after 10–12 months of treatment with first-generation EGFR-TKIs (33, 34) due to emergence of new EGFR mutations. For instance, the EGFR-T790M mutation enhances the affinity of its kinase domain for ATP, and lowers the efficacy of TKIs.

Second-generation EGFR-TKIs, such as afatinib, irreversibly bind to EGFR. In addition to inhibiting cells with TKI-sensitive EGFR mutations, second-generation TKIs are effective in some patients who are resistant to first-generation TKIs (35, 36). However, while afatinib shows more persistent inhibitory effect against the EGFR signaling pathway compared to other TKIs, it lacks selectivity and also inhibits the proliferation of normal cells. This off-target action of afatinib results in severe side effects such as diarrhea, rash, and oral mucositis, leading to treatment discontinuation in some patients (37, 38).

**Figure 2:**
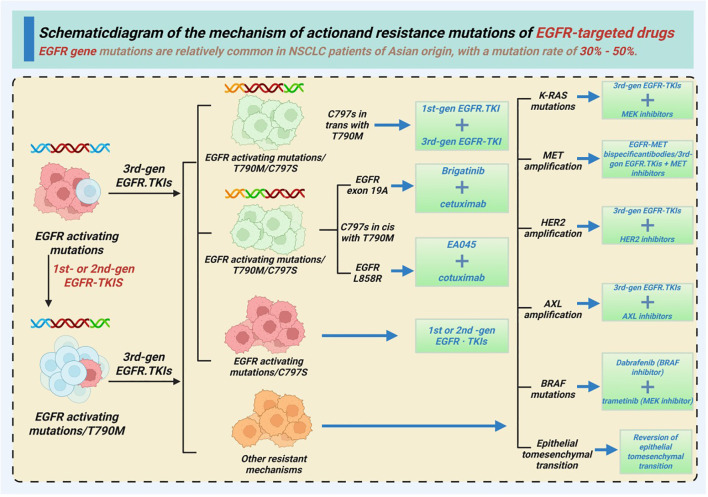
Schematic diagram of the mechanism of action of EGFR-targeted drugs and the EGFR mutations involved in drug resistance.

The third-generation EGFR-TKI osimertinib was developed to target EGFR-T790M drug-resistant mutation, and has shown good efficacy against drug-sensitive EGFR mutations (39–41). It can also penetrate the blood–brain barrier and effectively control brain metastases. Furthermore, osimertinib has better safety and tolerability due to its weak inhibitory effect on wild-type EGFR (42, 43). In the FLAURA study, osimertnib achieved a median PFS of 18.9 months as a first-line treatment for NSCLC patients with drug-sensitive EGFR mutations, and the survival benefit was superior compared to that of first-generation EGFR-TKIs. Recent studies have shown that some new EGFR inhibitors, such as poziotinib, are effective against EGFR with exon 20 insertion mutations, which are recalcitrant to traditional EGFR-TKIs (43, 44). The results of a clinical study showed that poziotinib effectively controlled tumor growth in patients with EGFR exon 20 insertion mutations. The mechanisms underlying the action of EGFR-targeted drugs and the drug-resistance mutations are shown in **Figure 2** (45–47).

EGFR mutations often trigger constitutive activation of the RAS-RAF-MEK-ERK and PI3K-AKT-mTOR pathways, often in concert with parallel receptor tyrosine kinases (RTKs) such as MET and HER2. Under the selective pressure of first- and third-generation EGFR-TKIs, tumor cells can adapt through on-target alterations (for example, T790M or C797S), as well as via bypass-track activation, such as MET amplification, HER2 or AXL upregulation, and reactivation of downstream signaling. These resistance loops provide a clear mechanistic rationale for rational combinatorial approaches. For instance, dual blockade of EGFR and MET may overcome MET-amplified or MET-driven resistance, whereas combining EGFR-TKIs with PD-1/PD-L1 inhibitors can potentially counteract TKI-induced upregulation of PD-L1 and the induction of an immunosuppressive tumor microenvironment (TME). Early-phase studies on EGFR-mutant NSCLC patients suggest that these drug combinations may deepen responses in selected patients, although optimization of patient selection, treatment sequencing, and toxicity management remain key areas for future research.

#### 2.1.2 Drugs targeting anaplastic lymphoma kinase fusion

ALK fusion is one of the most common genetic variations in NSCLC, and accounts for approximately 5% of all mutations. Fusion of the ALK gene leads to the continuous activation of the kinase, thus driving the growth and proliferation of tumor cells (48–50). The first-generation ALK inhibitor crizotinib competitively binds to the ATP-binding site of the kinase domain, and inhibits tumor growth by blocking the downstream signaling pathway (51, 52). Compared to traditional chemotherapy, crizotinib significantly improved the ORR and PFS of NSCLC patients harboring ALK fusion (ALK-positive), resulting in better prognosis. However, crizotinib resistance frequently develops during treatment (53, 54), and is mainly attributed with mutations in the kinase domain, increase in ALK gene copy number, and the activation of bypass signaling pathways (55–57).

Second-generation ALK inhibitors, including ceritinib, alectinib, and brigatinib, exhibit stronger inhibitory effect against ALK, and can partially overcome the mutations involved in crizotinib resistance. In addition, second-generation ALK inhibitors can effectively treat brain metastases due to increased blood-brain barrier penetration (58, 59). Alectinib has shown excellent intracranial antitumor activity in multiple experimental studies. In the ALEX study, first-line treatment with alectinib prolonged the median PFS of ALK-positive NSCLC patients to 34.8 months compared to 9.3 months in the crizotinib-treated arm. Furthermore, alectinib was also effective in patients with brain metastases. The third-generation ALK inhibitor lorlatinib can target multiple ALK mutations, and has a greater ability to penetrate the blood–brain barrier (26, 60). Lorlatinib has shown good efficacy in ALK-positive NSCLC patients resistant to crizotinib and second-generation ALK inhibitors. In the CROWN study, the median PFS (mPFS) of ALK-positive NSCLC patients treated with crizotinib was 9.3 months, whereas that of the Lorlatinib prolonged the PFS of patients (61–65).

#### 2.1.3 KRAS-targeted drugs

Activating KRAS mutations are present in approximately 25-30% of non-squamous NSCLCs – predominantly in lung adenocarcinomas. The frequency of these mutations is higher in Western cohorts compared to East Asian populations. The KRAS protein was considered “undruggable” for a long time due to its unique structure and the lack of suitable drug-binding sites (66–68). Since 2016, structural biology and medicinal chemistry have been applied to devise strategies for targeting KRAS mutations. Sotorasib and Adagrasib are KRAS mutant-targeting drugs that have been approved for commercial use (69, 70). Sotorasib covalently binds to the KRASG12C mutant and locks it in an inactivated state, thus blocking the downstream pathway. It has shown good efficacy and safety in NSCLC patients with the KRASG12C mutation. In the CodeBreaK100 study, sotorasib achieved an ORR of 37.1% in NSCLC patients with KRASG12C mutations, and prolonged the mPFS to 6.8 months. Adagrasib also targets the KRASG12C mutant, and has greater affinity and longer half-life compared to sotorasib, potentially resulting in a more sustained therapeutic effect. KRASG12C-positive NSCLC patients treated with adagrasib achieved 43% ORR, with mPFS of 6.5 months (71, 72). KRASG12C inhibitors can be combined with immune checkpoint inhibitors (ICIs), MEK inhibitors, etc. to improve their efficacy (73–75).

In summary, targeted therapies in lung cancer exemplify the paradigm of drugging specific oncogenic drivers such as EGFR, ALK, and KRAS, thereby providing substantial clinical benefit for well-defined molecular subgroups. Nevertheless, heterogeneous resistance mechanisms, limited benefit in oncogene-negative disease, and challenges in achieving durable central nervous system control still constrain long-term outcomes. These outcomes underscore the importance of pathway-directed strategies and resistance-aware drug development, themes that also underpin targeted approaches in other thoracic malignancies. In the following subsection, we therefore turn to breast cancer, where HER2 amplification and dysregulation of the PI3K-AKT-mTOR pathway have led to similarly transformative yet still incomplete advances in molecular-targeted therapy.

### 2.2 Breast cancer

**Figure 3:**
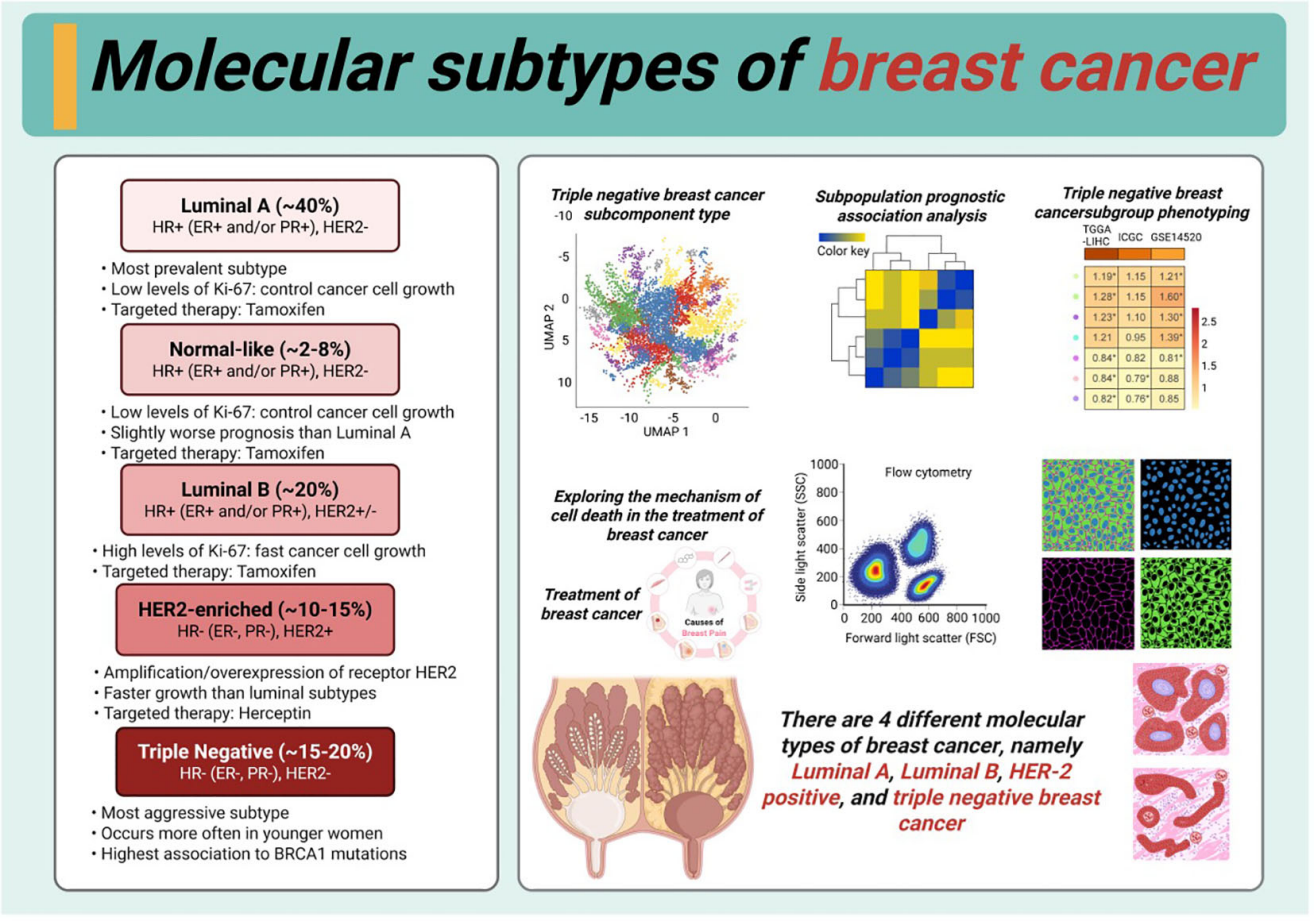
Molecular subtypes of breast cancer.

Breast cancer is one of the most common malignancies in women. According to its molecular characteristics, breast cancer can be divided into the luminal A, luminal B, human epidermal growth factor receptor (HER)2-overexpressing, and triple-negative subtypes (76–78) (**Figure 3**). Several targeted therapies have been developed for breast cancer in recent years (79, 80).

#### 2.2.1 HER2-targeted therapy

HER2, a member of the human epidermal growth factor receptor family, is often overexpressed or amplified in cancer cells, and promotes their proliferation, survival, and metastasis. HER2-positive breast cancer accounts for approximately 15% - 20% of all cases, and is characterized by highly invasive tumors and poor prognosis (81, 82). Trastuzumab is the first monoclonal antibody to be approved for the treatment of HER2-positive breast cancer. It binds to the extracellular domain of HER2, and inhibits its dimerization and autophosphorylation, thus blocking the downstream signaling pathway (83–85). In addition, trastuzumab can also eliminate cancer cells through antibody-dependent cell-mediated cytotoxicity (ADCC), and activate natural killer (NK) cells. The combination of trastuzumab and chemotherapy has been shown to improve the survival of HER2-positive breast cancer patients and reduce the risk of recurrence (86–88). A combined analysis of the NSABPB-31 and NCCTGN9831 studies revealed that trastuzumab combined with chemotherapy lowered the recurrence risk and mortality in HER2-positive breast cancer patients by 52% and 33% respectively (33, 89).

Pertuzumab binds to the dimerization domain of HER2 and prevents formation of heterodimers with other HER family members, resulting in complete inactivation of the HER2 signaling pathway (90–92). It can be used in combination with trastuzumab to further improve the treatment benefits in HER2-positive breast cancer. In the CLEOPATRA trial, HER2-positive advanced breast cancer patients treated with the combination of trastuzumab, pertuzumab, and docetaxel had a median overall survival (mOS) of 56.5 months, compared to 40.8 months reported in patients treated with trastuzumab and docetaxel (93, 94).

Trastuzumab emtansine (T-DM1) is an antibody-drug conjugate (ADC) consisting of trastuzumab and the cytotoxic drug mertansine. T-DM1 selectively delivers mertansine to HER2-positive tumor cells through trastuzumab, and the released drug inhibits tubulin polymerization and induces apoptosis (95, 96). In the EMILIA study involving HER2-positive advanced breast cancer patients, T-DM1 extended the median PFS to 9.6 months and the OS to 30.9 months, and resulted in milder adverse events than that observed with the combination of lapatinib and capecitabine (97–99).

#### 2.2.2 Drugs targeting the PI3K-AKT-mTOR pathway

The PI3K-AKT-mTOR pathway plays a key role in the proliferation, survival, and metabolism of breast cancer cells. Abnormal activation of this pathway is closely related to the occurrence, development, and drug resistance of breast cancer (100–102). Everolimus is an mTOR inhibitor that forms a complex with the intracellular FKBP12 protein, and blocks the downstream pathway (103, 104). The combination of everolimus and endocrine therapy can improve the PFS of patients with hormone receptor-positive and HER2-negative advanced breast cancer. In the BOLERO-2 study, the combination of everolimus and exemestane increased the ORR in postmenopausal women with hormone receptor-positive and HER2-negative advanced breast cancer to 12.6% compared with exemestane monotherapy (105–107), and extended the mPFS from 3.2 months to 7.8 months.

Collectively, targeted therapy in breast cancer illustrates how precise molecular subtyping can be translated into effective HER2-directed and pathway-focused regimens, while also revealing persistent obstacles such as primary and acquired resistance, treatment-related toxicity, and economic burden. These opportunities and limitations closely mirror those observed in lung cancer and highlight the need to extend rational targeted strategies to less common thoracic tumors. The next subsection therefore focuses on thymoma, a rare but immunologically distinctive malignancy in which early exploratory data suggest that EGFR-directed agents, recombinant monoclonal antibodies, and novel compounds such as Sitongzhi may open new avenues for patients who have historically lacked effective targeted options.

### 2.3 Thymoma

Thymomas are relatively rare thoracic tumors that originate from epithelial cells, and are difficult to treat due to the limited efficacy of conventional therapeutic strategies. Molecular targeted therapy offers a new direction for the treatment of thymoma (108–110).

#### 2.3.1 EGFR-targeted drugs

The growth and metastasis of thymoma cells is associated with the activation of the EGFR signaling pathway. Although EGFR-targeted drugs, such as erlotinib, can be effective against thymoma cells (111–113), their scope is limited due to the complex molecular and biological characteristics of thymomas (114, 115). Only a small proportion of thymoma patients with high EGFR expression may benefit from EGFR-targeted therapy. In small-scale clinical trials, erlotinib treatment led to tumor shrinkage in some EGFR^high^ thymoma patients, although the overall response rate was not high (116–118).

#### 2.3.2 Recombinant monoclonal antibody drugs

Recombinant monoclonal antibody drugs, including targeted drugs and ICIs, can inhibit the growth and metastasis of thymoma cells through multiple mechanisms by specifically binding to surface antigens (119, 120). Monoclonal antibodies targeting tumor-specific antigens can trigger ADCC and activate the cytotoxic immune effector cells to kill tumor cells. ICIs targeting programmed death receptor 1 (PD-1) and its ligand (PD-L1) can relieve the inhibitory effects of tumor cells on the immune system and restore anti-tumor immune responses (24, 121, 122). PD-1 inhibitors have shown efficacy in thymoma patients, and achieved stable disease in some patients.

#### 2.3.3 Sitongzhi

Sitongzhi inhibits the growth and metastasis of tumor cells, promotes the proliferation and activation of T cells (123–125), and enhances the recognition and killing of thymoma cells by T cells. Furthermore, it can also improve the tumor microenvironment (TME) and inhibit angiogenesis, resulting in further tumor inhibition. Although Sitongzhi has demonstrated anti-tumor activity in animal models of thymoma, its efficacy and safety in humans need to be further verified by larger clinical studies (126, 127). A concise overview of representative targeted therapy trials in thoracic tumors has been provided in **Table 1**.

**Table 1:** Selected landmark trials of targeted therapy in thoracic tumors.

Cancer type	Trial/study	Agent(s) (experimental vs control)	Line of therapy	Population (key inclusion)	Key efficacy outcomes (median PFS/OS, ORR)	Headline toxicity profile
NSCLC	FLAURA	Osimertinib vs first-generation EGFR-TKIs	First-line	Advanced EGFR-sensitizing mutant NSCLC	Osimertinib achieved a median PFS of 18.9 months and significantly improved OS compared with first-generation EGFR-TKIs.	Class-related EGFR-TKI AEs (rash, diarrhea) with an overall improved tolerability profile versus first-generation EGFR-TKIs.
NSCLC	ALEX	Alectinib vs crizotinib	First-line	Advanced ALK-positive NSCLC	Median PFS 34.8 vs 9.3 months (alectinib vs crizotinib), with superior intracranial disease control in the alectinib arm.	Similar overall AE spectrum; lower incidence of some crizotinib-associated toxicities and favorable CNS safety with alectinib.
NSCLC	CROWN	Lorlatinib vs crizotinib	First-line	Advanced ALK-positive NSCLC	Median PFS 9.3 months in the crizotinib group; median PFS not reached for lorlatinib at analysis, with markedly improved intracranial efficacy.	Higher rates of hyperlipidemia and CNS-related AEs with lorlatinib, generally manageable with monitoring and dose modification.
NSCLC	CodeBreaK 100	Sotorasib (single-arm)	Later-line (post-standard therapy)	Advanced NSCLC with KRAS G12C mutation	ORR 37.1%; median PFS 6.8 months in a heavily pretreated population.	Mostly low-grade gastrointestinal and hepatic AEs; treatment discontinuation due to toxicity was relatively uncommon.
NSCLC	KRYSTAL-1 (NSCLC cohort)	Adagrasib (single-arm)	Later-line	Advanced NSCLC with KRAS G12C mutation	ORR 43%; median PFS 6.5 months, demonstrating meaningful activity in a molecularly defined, previously treated population.	Gastrointestinal AEs (nausea, diarrhea) and fatigue most common, generally manageable with supportive care.
Breast cancer	CLEOPATRA	Trastuzumab + pertuzumab + docetaxel vs trastuzumab + docetaxel	First-line	HER2-positive metastatic/advanced breast cancer	Median OS 56.5 vs 40.8 months and prolonged PFS for the triplet regimen compared with trastuzumab + docetaxel; higher ORR.	Higher rates of diarrhea and neutropenia in the triplet arm, but AEs were acceptable and manageable.
Breast cancer	EMILIA	Trastuzumab emtansine (T-DM1) vs lapatinib + capecitabine	Second-line and beyond	Previously treated HER2-positive advanced breast cancer	T-DM1: median PFS 9.6 months, median OS 30.9 months; significantly better outcomes than lapatinib + capecitabine.	Lower incidence of severe diarrhea and hand–foot syndrome; thrombocytopenia and elevated liver enzymes are the main toxicities.
Breast cancer	BOLERO-2	Everolimus + exemestane vs exemestane alone	Endocrine-resistant, postmenopausal	HR-positive, HER2-negative advanced breast cancer	Combination: ORR 12.6%; median PFS 7.8 vs 3.2 months compared with exemestane alone.	Stomatitis, non-infectious pneumonitis and metabolic abnormalities more frequent, but usually manageable with dose adjustment and supportive care.
Thymoma	Phase II/pilot studies	Erlotinib or other EGFR-TKIs (single-arm)	Refractory/relapsed	EGFR-high thymoma progressing after standard therapy	Partial responses and disease stabilization in a subset of patients; overall response rates modest but clinically meaningful.	AEs consistent with EGFR-TKI class (rash, diarrhea); severe toxicities infrequent in small available cohorts.

## 3 New progress in immunotherapy

Immunotherapy encompasses several complementary strategies that activate the anti-tumor immune response. In this section, we have summarized the current status of ICIs across major thoracic malignancies, and discussed emerging approaches such as adoptive transfer of tumor-infiltrating lymphocytes (TILs) and individualized cancer vaccines. Together, these modalities illustrate how modulation of immune checkpoints, effector cells, and antigen presentation can be leveraged to improve outcomes in thoracic tumors.

### 3.1 Immune checkpoint inhibitors

**Figure 4:**
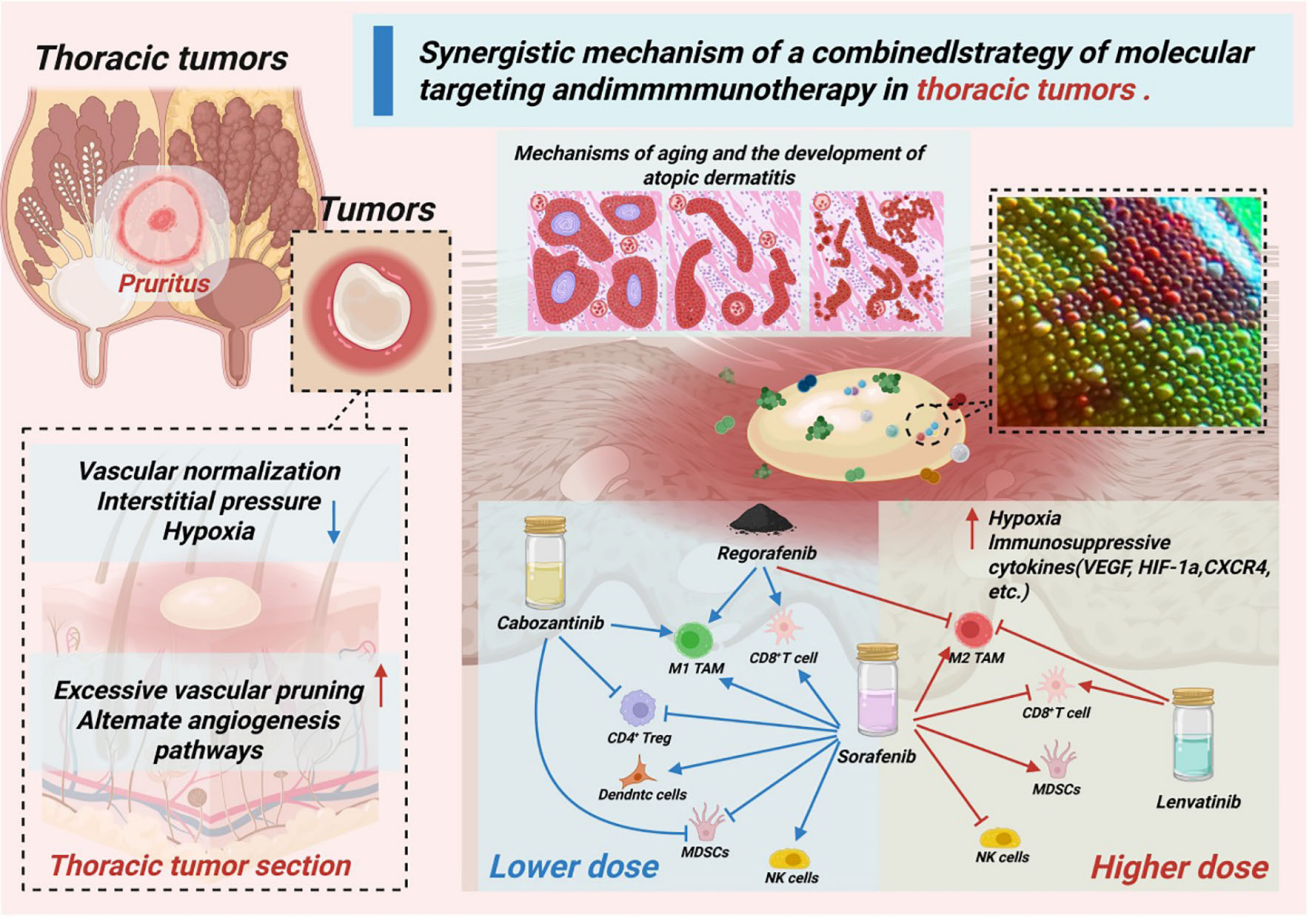
Mechanism of action of ICIs.

Immune checkpoints like PD-1, PD-L1, and cytotoxic T lymphocyte-associated antigen 4 (CTLA-4) are a group of regulatory molecules that limit the damage caused by immune responses to normal tissues, and are also used by tumor cells to escape immune surveillance (128–130). ICIs can relieve tumor-induced immunosuppression by disrupting the interaction between immune checkpoints and their ligands, and restore the ability of T cells to eliminate tumor cells (**Figure 4**) (131, 132).

In addition to the clinically validated PD-1/PD-L1 and CTLA-4 pathways (**Figure 4**), inhibitory receptors such as T cell immunoreceptor with Ig and ITIM domains (TIGIT), lymphocyte activation gene-3 (LAG-3), and T cell immunoglobulin and mucin-domain containing-3 (TIM-3) have been implicated in T-cell exhaustion in thoracic malignancies. Co-expression of these molecules with PD-1 often marks deeply dysfunctional T-cell populations in NSCLC, breast cancer and esophageal cancer, and preclinical studies suggest that dual or triple blockade can further invigorate immune responses against tumors that are recalcitrant to PD-1/PD-L1 monotherapy. Multiple phase I/II trials are therefore evaluating antibodies against TIGIT, LAG-3 or TIM-3 alone or in combination with PD-1/PD-L1 inhibitors in the treatment of lung and other thoracic tumors. Although most of these agents have not yet been integrated into routine clinical practice, they represent a next wave of ICIs that may expand the population benefiting from immunotherapy, and help overcome acquired resistance.

#### 3.1.1 Lung cancer

Pembrolizumab monotherapy is currently used to treat advanced NSCLC patients with high PD-L1 expression (TPS ≥ 50%). The KEYNOTE-024 study revealed that, under second-line conditions, first-line treatment of PD-L1^high^ patients with pembrolizumab extended the mOS from 22.1 months to 30 months, and the mPFS from 6 months to 10.3 months compared to traditional chemotherapy (133–135). Furthermore, the adverse reactions of prembolizumab were relatively mild. The combination of PD-L1/PD-L1 inhibitors and chemotherapy also showed good curative effect in the PD-L1^low/-^ patients (136, 137). In the KEYNOTE-189 study, pembrolizumab combined with pemetrexed and platinum-based chemotherapy increased the ORR of lung cancer patients from 29.4% to 47.6% compared to chemotherapy, and extended the mPFS from 4.9 months to 8.8 months, and the mOS from 12 months to 22 months (138–140).

ICIs, particularly PD-1 blockers, have been incorporated into the standard treatment for SCLC. The IMpower133 study showed that atezolizumab combined with etoposide and platinum-based chemotherapy extended the mOS of SCLC patients from 10.3 months to 12.3 months, and the mPFS from 4.3 months to 5.2 months compared with chemotherapy alone. The bispecific antibody drug tarlatamab targets the T cell-specific CD3 and the DLL3 protein, which is often overexpressed on SCLC cells. Thus, simultaneous binding of tarlatamab to CD3 and DLL3 allows T cells to aggregate around the tumor cells, resulting in selective elimination of the latter (115, 141, 142). A preliminary clinical trial showed that tarlatamab can control tumor growth in some SCLC patients.

#### 3.1.2 Breast cancer

Immunotherapy has also proven beneficial for triple-negative breast cancer (TNBC) patients. In a clinical study based on the “Fudan classification”, the combination of albumin-bound paclitaxel, the PD-1 blocker camrelizumab, and the TKI famitinib prolonged the PFS of patients with metastatic TNBC to 15.1 months (143–145), an increase of 8.6 months compared to traditional chemotherapy, and achieved an ORR of 80% (146, 147). In the KEYNOTE-355 study conducted on PD-L1-positive (CPS≥10) metastatic TNBC patients, pembrolizumab combined with chemotherapy extended the PFS from 5.6 months to 9.7 months, and the OS from 16.1 months to 23 months (148–150).

#### 3.1.3 Other thoracic tumors

The therapeutic effects of ICIs have been demonstrated in large-scale clinical studies involving esophageal cancer and thymoma patients. The combination of the PD-1 blocker nivolumab and chemotherapy is currently among the first-line treatment options for patients with advanced esophageal cancer. The ATTRACTION-5 study confirmed that compared to chemotherapy alone, nivolumab combined with chemotherapy can reduce the mortality risk of patients with advanced esophagogastric junction cancer by 25%, and increase the mOS from 10.9 months to 12.6 months (151–153). In addition, pembrolizumab alone or in combination with chemotherapy also offers new possibilities for esophageal cancer patients. In the KEYNOT-590 study, pembrolizumab combined with chemotherapy for first-line treatment of advanced esophageal cancer or esophagogastric junction cancer significantly improved the ORR and OS of patients compared to chemotherapy alone.

ICIs have proven to be an effective therapeutic option for thymomas (154–156). Some thymoma patients have experienced tumor remission and stable disease following treatment with PD-1/PD-L1 inhibitors. However, due to the heterogenous immune microenvironment of thymomas, many patients develop primary or secondary resistance to ICIs, resulting in sub-optimal treatment outcomes. Therefore, it is crucial to elucidate the immune escape mechanism of thymomas, screen for effective biomarkers (157), select suitable patients, and devise combination treatment strategies in order to improve the efficacy of immunotherapies.

A non-exhaustive overview of key ICIs that have been approved or are guideline-recommended for major thoracic malignancies is presented in **Table 2**.

**Table 2:** Selected ICIs approved or guideline-recommended for thoracic malignancies.

Thoracic tumor type	Immune checkpoint inhibitor (examples)	Checkpoint target	Typical indication/regimen (representative setting)	Representative pivotal studies mentioned in this review
Non-small cell lung cancer (NSCLC)	Pembrolizumab	PD-1	First-line monotherapy for advanced/metastatic NSCLC with high PD-L1 expression (TPS ≥ 50%); pembrolizumab plus platinum-based chemotherapy for advanced non-squamous NSCLC regardless of PD-L1 status	KEYNOTE-024; KEYNOTE-189
Small cell lung cancer (SCLC)	Atezolizumab	PD-L1	First-line treatment of extensive-stage SCLC in combination with etoposide and platinum-based chemotherapy	IMpower133
Triple-negative breast cancer (TNBC)	Pembrolizumab; camrelizumab (region-specific)	PD-1	Pembrolizumab plus chemotherapy for PD-L1-positive (CPS ≥ 10) metastatic or unresectable TNBC; combinations of PD-1 blockade with taxane-based chemotherapy and TKIs explored in metastatic TNBC	KEYNOTE-355; Fudan classification-based trial cited in text
Esophageal/esophagogastric junction cancer	Nivolumab; pembrolizumab	PD-1	Nivolumab plus chemotherapy as first-line therapy for advanced esophageal or esophagogastric junction cancer; pembrolizumab plus chemotherapy as first-line treatment in PD-L1-expressing advanced disease	ATTRACTION-5; KEYNOTE-590
Thymoma	PD-1/PD-L1 inhibitors (e.g., pembrolizumab, nivolumab)	PD-1/PD-L1	Relapsed or refractory thymoma after standard therapy; used mainly in single-arm phase II studies and early-phase trials, with partial responses and disease stabilization in a subset of patients	Multiple small-scale clinical studies referenced in section 3.1.3

Taken together, clinical experience with PD-1/PD-L1 and CTLA-4 blockade in lung cancer, breast cancer, esophageal cancer, and thymoma has established immune checkpoint inhibition as a cornerstone of systemic therapy for thoracic malignancies. At the same time, primary and acquired resistance, heterogeneous PD-L1 expression, and immune-related adverse events (isAEs) limit the proportion of patients who derive durable benefit. These limitations have stimulated interest in complementary immunotherapeutic strategies, including cellular therapies and vaccines, which may broaden the spectrum of responders or be combined with ICIs to enhance efficacy. The major landmark immunotherapy trials across thoracic tumor types are summarized in **Table 3**.

**Table 3:** Selected landmark immunotherapy trials in thoracic tumors.

Cancer type	Trial/study	Agent(s) (experimental vs control)	Line of therapy	Population (key inclusion)	Key efficacy outcomes (median PFS/OS, ORR)	Headline toxicity profile
NSCLC	KEYNOTE-024	Pembrolizumab vs platinum-based chemotherapy	First-line	Advanced NSCLC without targetable drivers, PD-L1 TPS ≥ 50%	Median PFS 10.3 vs 6.0 months and median OS 30.0 vs 22.1 months; higher ORR with pembrolizumab.	Immune-related AEs (thyroid dysfunction, pneumonitis, skin reactions); overall better tolerability and fewer high-grade AEs than chemotherapy.
NSCLC	KEYNOTE-189	Pembrolizumab + pemetrexed + platinum vs chemotherapy alone	First-line	Advanced non-squamous NSCLC, any PD-L1 status	ORR 47.6% vs 29.4%; median PFS 8.8 vs 4.9 months; median OS 22 vs 12 months (pembrolizumab combo vs chemotherapy).	Increased immune-related AEs compared with chemotherapy alone, but toxicity was generally manageable; no major new safety signals.
SCLC	IMpower133	Atezolizumab + etoposide + platinum vs chemotherapy alone	First-line	Extensive-stage SCLC	Median OS 12.3 vs 10.3 months; median PFS 5.2 vs 4.3 months (atezolizumab combo vs chemotherapy); survival benefit with PD-L1 blockade.	Safety profile consistent with chemotherapy plus PD-L1 inhibition; immune-related toxicities manageable with standard algorithms.
TNBC	Fudan-based trial (albumin-bound paclitaxel + camrelizumab + famitinib)	Albumin-bound paclitaxel + camrelizumab + famitinib vs chemotherapy regimen	First-line/early-line for metastatic TNBC	Metastatic TNBC, selected by Fudan molecular classification	Combination: median PFS 15.1 months (≈8.6-month improvement vs chemotherapy), ORR around 80%.	Mainly hematologic and immune-related toxicities (e.g. neutropenia, hypertension, endocrinopathies), generally manageable with supportive care and dose modification.
TNBC	KEYNOTE-355	Pembrolizumab + chemotherapy vs chemotherapy alone	First-line	Metastatic or unresectable TNBC, PD-L1 CPS ≥ 10	PFS 9.7 vs 5.6 months and OS 23.0 vs 16.1 months (pembrolizumab combo vs chemotherapy); improved ORR and durable responses.	More immune-related AEs (thyroid disorders, hepatitis, skin AEs) than chemotherapy alone; overall acceptable and consistent with known pembrolizumab safety.
Esophageal/EGJ cancer	Nivolumab + chemotherapy trial (e.g. ATTRACTION-type)	Nivolumab + chemotherapy vs chemotherapy alone	First-line	Advanced esophageal or esophagogastric junction cancer	Nivolumab combination reduced the risk of death and increased median OS compared with chemotherapy; improved ORR and PFS.	Toxicity profile similar to chemotherapy plus PD-1 blockade; immune-related AEs (rash, hepatitis, pneumonitis) generally manageable.
Esophageal/EGJ cancer	KEYNOTE-590	Pembrolizumab + chemotherapy vs chemotherapy alone	First-line	Advanced esophageal or EGJ cancer, with emphasis on PD-L1–positive subsets	Significant improvement in OS and ORR with pembrolizumab plus chemotherapy vs chemotherapy alone, particularly in PD-L1–expressing patients; PFS also prolonged.	Immune-related toxicities typical of PD-1 blockade (thyroid abnormalities, rash, pneumonitis); overall acceptable and manageable safety profile.
Thymoma	Phase II/early-phase PD-1/PD-L1 inhibitor trials	Pembrolizumab or nivolumab (mainly single-arm or small combinations)	Later-line (post-chemotherapy)	Relapsed or refractory thymoma	Partial responses and durable stable disease in a subset; modest overall response rates but clinically meaningful in heavily pretreated patients.	Immune-related AEs can be relatively frequent and severe (e.g. autoimmune myocarditis, myositis); require close monitoring and prompt immunosuppressive management.

### 3.2 Adoptive transfer of tumor-infiltrating lymphocytes

TILs persist in the TME for a long time, and can recognize and kill tumor cells. The TILs isolated from tumors can be expanded *in vitro* and reinfused into the patient to initiate an anti-tumor immune response. The adoptive transfer of TILs presents a promising approach for the treatment of lung cancer. A significant proportion of lung cancer patients have experienced tumor shrinkage and prolonged survival after receiving TIL infusion. In a clinical trial conducted by the National Cancer Institute of the United States, TIL infusion achieved a significant ORR in NSCLC patients, with acceptable safety (158–160). Nevertheless, there are several challenges associated with the clinical translation of TILs. The extraction and *in vitro* expansion of TILs are technically demanding, and the treatment is highly complex and expensive at present. Moreover, the sub-optimal infiltration and activity of TILs in tumor tissues, and their potential adverse reactions are other challenges that need to be overcome.

Beyond TIL products, other cell-based immunotherapies are being actively explored in thoracic oncology. NK cell-based strategies, including autologous and allogeneic infusions, as well as genetically modified chimeric antigen receptor (CAR)-NK products, offer the potential for potent cytotoxicity with a lower risk of graft-versus-host disease and cytokine release syndrome. Early-phase studies in NSCL and SCLC patients have demonstrated acceptable safety profiles and preliminary signals of anti-tumor activity. CAR-T cell therapies directed against HER2, mesothelin and EGFR are also under investigation in thoracic tumors; however, on-target/off-tumor toxicities, limited trafficking into solid tumor sites, and the highly immunosuppressive TME remain major obstacles. As manufacturing platforms and safety-engineering strategies continue to improve, these cellular therapies may gradually move from proof-of-concept toward broader clinical application in carefully selected patients with advanced thoracic malignancies.

### 3.3 Cancer vaccines

Tumor vaccines induce specific immunity against tumor cells, and can be further divided into tumor-preventive and anti-tumor vaccines. The combined application of some individualized anti-tumor vaccines and ICIs has reduced the recurrence risk in lung cancer patients (161, 162). Individualized vaccines are designed on the basis of tumor mutations, and may induce an immune response against tumor-specific antigens. In one study, an individualized preventive lung cancer vaccine was successfully used in combination with ICIs. Furthermore, vaccination of patients with metastatic lung cancer after tumor surgery reduced the risk of recurrence and extended the recurrence-free survival. However, most tumor vaccines are currently in the clinical trial stage, and their clinical translation will depend on overcoming challenges such as immunogenicity (163–165), safety, and tumor immune escape. In addition, the production of vaccines is technically complex and costly, which hinder their large-scale clinical application. Thus, the future directions for the development of individualized tumor vaccines would be optimization of design and production process, and cost-control (77, 166, 167).

Overall, advances in immune checkpoint blockade, TIL therapy, and personalized cancer vaccines demonstrate the versatility of immunotherapy platforms for thoracic tumors, but also reveal practical constraints related to manufacturing complexity, cost, and patient selection. The next step is to integrate these immune-based approaches with molecular-targeted agents that reshape oncogenic signaling and the TME. In the following section, we therefore focus on rational combination strategies that couple targeted therapy with immunotherapy to achieve more durable and synergistic clinical benefits.

## 4 Combined strategies of molecular targeted therapy and immunotherapy

**Figure 5:**
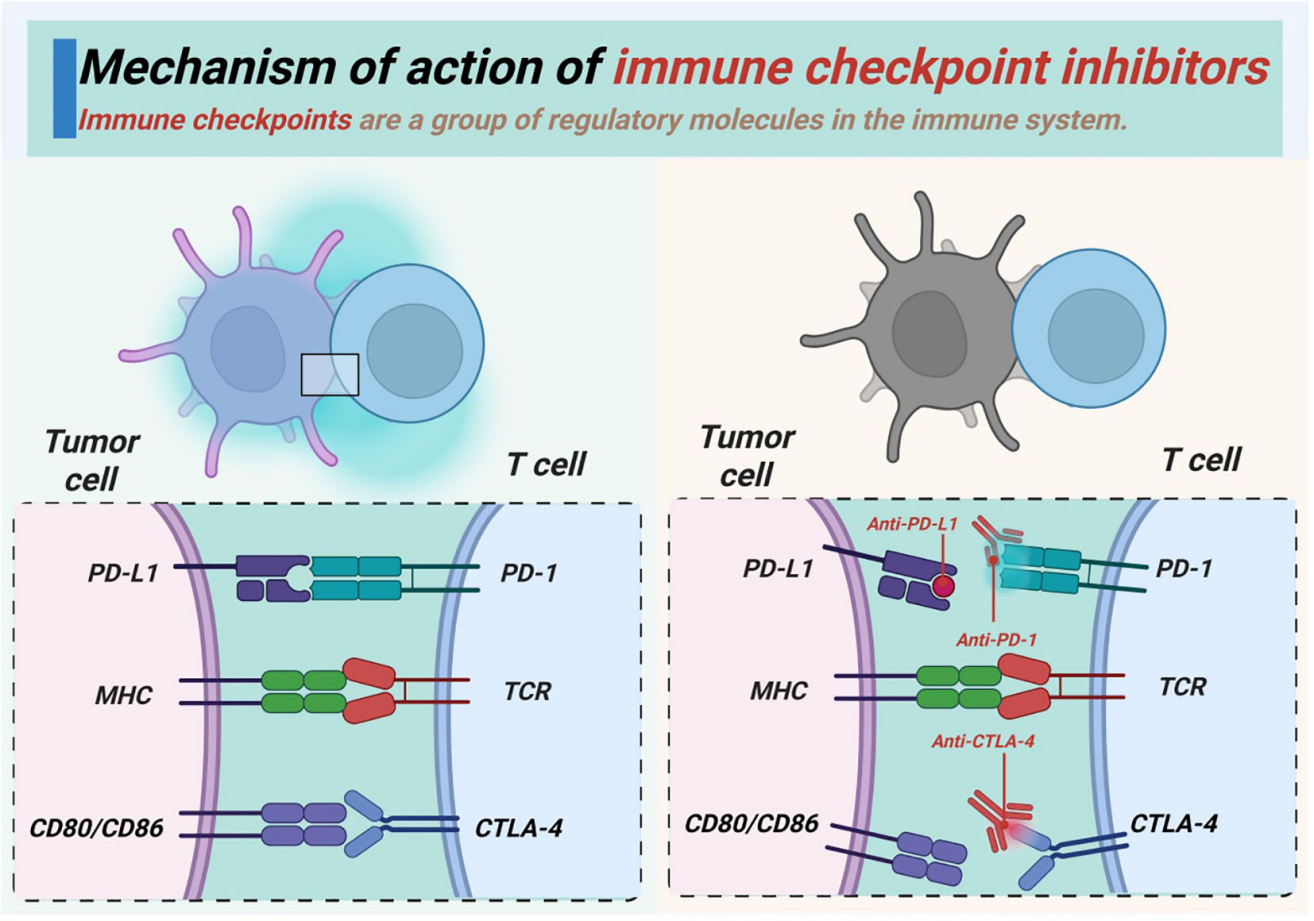
Synergistic action of molecular targeting and immunotherapy against thoracic tumors.

Immunotherapy and molecular targeted therapy can achieve effective tumor control through synergistic action. While molecular targeted therapy can specifically inhibit tumor growth and proliferation (168–170), remodel the TME, and increase the immunogenicity of tumor cells, immunotherapy can activate the immune system and kill tumor cells (**Figure 5**).

### 4.1 Lung cancer

The combination of EGFR-TKIs and PD-1 inhibitors offers unique advantages in the treatment of lung cancer. The cellular stress induced by the EGFR-TKIs in EGFR-positive lung tumors triggers the release of tumor-associated antigens (171–173). These antigens are captured and presented to T cells by antigen-presenting cells, thereby increasing tumor immunogenicity and augmenting the effect of PD-1 inhibitors. However, some patients experience a relatively high incidence of adverse reactions after receiving this combination treatment (174–176). This may be due to the complex effects of EGFR-TKIs on the immune microenvironment, along with increased immune activation through PD-1 blockade. It will be a topic of future research to achieve satisfactory efficacy with this combination therapy while reducing adverse reactions. The combination of ALK inhibitors and ICIs has also shown therapeutic effects in preliminary clinical trials (177–179). ALK inhibitors can enhance the immunogenicity of ALK-positive lung cancer cells by altering the surface antigens, which can potentially improve the efficacy of ICIs when used in combination.

However, most EGFR–PD-1/PD-L1 and ALK–ICI combinations have been evaluated only in early-phase, non-randomized trials, or in small retrospective cohorts involving limited numbers of patients. Although these studies provide preliminary indications of antitumor activity and feasibility, they are not sufficient to demonstrate superiority over established sequential targeted therapy or immunotherapy alone. Consequently, such regimens should be regarded as exploratory and preferably implemented within the context of clinical trials until more robust randomized data become available.

### 4.2 Breast cancer

HER2-positive breast tumors are highly immunogenic, and HER2-targeted drugs (such as trastuzumab) can not only inhibit the growth of tumor cells but also activate immune cells through the ADCC effect (180–182). Therefore, combining HER2 inhibitors with ICIs can potentially trigger a strong immune response against HER2-positive breast cancer cells. For instance, preliminary data shows that the combination of trastuzumab and pembrolizumab can improve the ORR and PFS in patients with HER2-positive advanced breast cancer compared to either monotherapy. In addition, the combination of PI3K-AKT-mTOR pathway inhibitors and immunotherapy has shown therapeutic efficacy against TNBC (183–185). Abnormal activation of the PI3K-AKT-mTOR pathway in the tumor cells aids in immune escape. The mTOR inhibitors like everolimus can reverse this immune escape, increase the infiltration and cytotoxic function of anti-tumor immune cells, and synergize with ICIs. It should be emphasized that evidence supporting HER2-ICI and PI3K-AKT-mTOR-ICI combinations in breast cancer is derived predominantly from phase I/II studies and small single-arm cohorts, and no large randomized trial has yet established a clear survival advantage of these regimens over current standard-of-care treatments.

### 4.3 Thymoma

The combination of molecular targeted therapy and immunotherapy is a promising approach for treating thymomas owing to their unique immune microenvironment (186–188). EGFR-targeted drugs and PD-1 inhibitors can not only reduce the proliferation of thymoma cells by inhibiting the EGFR signaling pathway but also relieve the immunosuppressive microenvironment and improve anti-tumor immune responses. The combination of new targeted drugs such as Sitongzhi and immunotherapy can also increase the ability of T cells to kill thymoma cells through the remodeling of the TME, thereby improving clinical efficacy. Currently, most of these combined treatments are in the clinical testing stage, and their efficacy and safety still need to be verified by large-scale, multicenter trials. The clinical evidence for targeted therapy-immunotherapy combinations in thymoma is limited to case series and early-phase studies with relatively small sample sizes, often involving only a few dozen patients. These results should therefore be interpreted as exploratory, and no firm conclusions regarding superiority over conventional approaches can yet be drawn. Second (189–191), the optimal combination drugs, drug dosages, and treatment sequences will also have to be standardized.

There are certain challenges in combining molecular targeted drugs and immunotherapy in the treatment of thoracic tumors. First, due to individual differences in the sensitivity to combination treatment, it will be crucial to screen for patients who can benefit the most from the treatment, and devise individualized treatments (192–194). Second, combination treatment can lead to an increase in adverse reactions, which will have to be minimized while improving therapeutic efficacy in order to improve the quality of life of patients (195–197). Third, the high costs of combined treatments limit their clinical application, thereby warranting strategies to reduce costs and improve accessibility.

## 5 Challenges and prospects

### 5.1 Drug resistance

A small fraction of patients either do not respond to targeted drugs or immunotherapy, or develop drug resistance. The mechanisms of drug resistance usually involve molecular-level adaptive changes in tumor cells under selection pressure. In addition to the definitive lung cancer-associated T790M mutation in the EGFR gene, new drug-resistant mutations such as C797S can lead to the failure of existing targeted drugs. Increased infiltration of immunosuppressive cells such as regulatory T cells and myeloid-derived suppressor cells in the TME can help tumor cells escape immune surveillance, and render them insensitive to immunotherapy. The heterogeneity of tumor cells is also a driving factor for drug resistance. Different tumor cell subsets differ in their drug sensitivities, and the expansion of drug-resistant clones during treatment leads to treatment failure. Therefore, a greater understanding of the mechanisms of drug resistance, development of new targeted drugs and immunotherapy strategies, and novel combination treatment plans will be key to overcoming drug resistance.

In clinical practice, several actionable strategies are being developed to translate mechanistic insights on resistance into dynamic management. Liquid biopsy-based monitoring of circulating tumor DNA (ctDNA) allows serial detection of emergent resistance alterations, such as secondary EGFR or ALK mutations and MET amplification, often before radiographic progression. Timely identification of these molecular events can guide therapeutic decisions, including switching to next-generation TKIs, adding targeted agents against bypass pathways, or redesigning combination regimens. For patients receiving immunotherapy, longitudinal ctDNA profiling and minimal residual disease assessment may help distinguish true progression from pseudo-progression and identify early molecular escape, thereby supporting risk-adapted continuation, intensification or de-escalation of treatment. Integrating these liquid biopsy approaches with tumor re-biopsy, advanced imaging and multi-omics profiling is expected to form a more precise and proactive framework for resistance surveillance in thoracic tumors.

### 5.2 Immune-related adverse reactions

Immunotherapies can cause immune-related adverse reactions in the skin, gastrointestinal tract, liver, endocrine system, and other organs, and seriously affect the quality of life of patients and may even be lethal. Therefore, a key challenge in this field is to better predict, monitor, and manage adverse reactions, and improve patient tolerance and quality of life. In the context of clinical application, it is necessary to establish a good adverse reaction monitoring system, identify biomarkers to predict the risk of adverse reactions, implement precise individualized treatment, adjust the treatment plan in a timely manner, and reduce the severity of adverse reactions. In addition, strengthening health education and improving patients’ compliance with self-monitoring of adverse reactions are also helpful for timely detection and treatment of adverse reactions.

Looking forward, a key direction is the development of predictive models that can stratify the risk of irAEs before and during treatment. Emerging evidence suggests that baseline clinical characteristics, serum biomarkers (such as cytokines, autoantibodies and organ-specific enzymes), host genomic factors (for example, HLA genotypes) and gut microbiota may collectively influence susceptibility to severe toxicity during immune checkpoint blockade. Machine learning-based approaches are increasingly being used to integrate these heterogeneous data and generate individualized risk scores for irAEs. In parallel, standardized algorithms for real-time toxicity monitoring, such as incorporating electronic patient-reported outcomes, regular laboratory testing and imaging where appropriate, can support earlier detection, graded intervention and coordinated multidisciplinary management. The implementation and prospective validation of such predictive and monitoring tools will be essential to maximize the therapeutic window of immunotherapy in thoracic oncology.

### 5.3 Prospects

Molecular typing of tumors through multiomics can help screen more patients who are likely to benefit from molecular targeted therapy and immunotherapy, and aid in devising individualized treatment. In-depth analysis of tumor cell heterogeneity via single-cell sequencing technology will provide more accurate targets for precision treatment. In addition, liquid biopsy can dynamically monitor the molecular changes in tumors, thus allowing clinicians to adjust the treatment plan. Moreover, continuous development of targeted therapy drugs and immunotherapy drugs, more effective combination treatment methods, and in-depth research on tumor immune escape mechanisms will lead to more breakthroughs in the treatment of thoracic tumors. The application of AI and big data will further accelerate drug development, and improve clinical decision-making and treatment efficacy. Large-scale clinical trials and international collaboration will be crucial to the progression of thoracic tumor treatment.

### 5.4 Multi-omics and AI for biomarker discovery and response prediction

The rapid advances in high-throughput technologies have enabled profiling of thoracic tumors at multiple molecular layers, such as the genome, transcriptome, epigenome, proteome, metabolome, and even radiome, which can detect clinically relevant heterogeneity that is not captured by single biomarkers. By integrating these multi-omics datasets, recent studies have identified composite signatures of NSCLC that predict clinical outcomes of immunotherapy more accurately than individual markers such as PD-L1 or tumor mutational burden. For example, Mei et al. combined genomic, transcriptomic, proteomic and ctDNA-derived features to build AI-driven risk models that stratify NSCLC patients receiving ICIs into distinct prognostic groups (198). Furthermore, multi-omics-driven machine learning can also delineate immune-inflamed versus immune-excluded phenotypes, identify therapy-responsive TME sub-cohorts, and suggest rational combinations of targeted agents and immunotherapies across solid tumors (199). In parallel, multi-modal models that fuse molecular, imaging and clinicopathological data are being developed to predict EGFR genotype and screen patients who can benefit from targeted therapy, further blurring the boundary between “molecular” and “imaging” biomarkers in thoracic oncology (200).

AI methods, including classical machine learning and deep learning, are central to extracting actionable information from these complex datasets and translating it into clinically usable tools. Deep learning radiomics models based on CT or PET/CT have been shown to infer PD-L1 status in a non-invasive manner, and predict durable benefit from immunotherapy in advanced NSCLC, thus supporting treatment decision-making tissues are limited or repeated biopsies are impractical (201). At the same time, longitudinal liquid-biopsy studies demonstrate that dynamic changes in circulating tumor DNA (ctDNA) can anticipate response, resistance and even hyper-progression under immune checkpoint blockade, and AI-enhanced analysis of serial ctDNA profiles is emerging as a powerful approach for real-time response monitoring (202). Systematic evaluations indicate that AI models for biomarker prediction in lung cancer can achieve robust sensitivity and specificity across heterogeneous cohorts, but also highlight key challenges, including data quality and harmonization, external validation, model interpretability and regulatory and ethical considerations. Overall, the convergence of multi-omics profiling, liquid biopsy and AI-assisted analytics is expected to become a major driving force behind biomarker discovery, individualized selection of targeted and immune therapies, and adaptive response prediction in thoracic tumors.

## 6 Conclusion

Molecular targeted therapy and immunotherapy have led to significant improvement in the prognosis and quality of life of patients with thoracic tumors. New targeted drugs and immunotherapy strategies have been developed in recent years for lung cancer, breast cancer, and thymoma, which also offer the possibility of combined application. However, despite their advantages, emergence of drug resistance and adverse reactions are pressing challenges. Nevertheless, molecular targeted therapy and immunotherapy will likely play a greater role in the treatment of thoracic tumors in the foreseeable future.
